# Structural Insight into Epitopes in the Pregnancy-Associated Malaria Protein VAR2CSA

**DOI:** 10.1371/journal.ppat.0040042

**Published:** 2008-02-15

**Authors:** Pernille Andersen, Morten A Nielsen, Mafalda Resende, Thomas S Rask, Madeleine Dahlbäck, Thor Theander, Ole Lund, Ali Salanti

**Affiliations:** 1 Center for Biological Sequence Analysis, BioCentrum-DTU, Denmark; 2 Centre for Medical Parasitology at University of Copenhagen and Copenhagen University Hospital (Rigshospitalet), Copenhagen, Denmark; Cornell University, United States of America

## Abstract

Pregnancy-associated malaria is caused by Plasmodium falciparum malaria parasites binding specifically to chondroitin sulfate A in the placenta. This sequestration of parasites is a major cause of low birth weight in infants and anemia in the mothers. VAR2CSA, a polymorphic multi-domain protein of the PfEMP1 family, is the main parasite ligand for CSA binding, and identification of protective antibody epitopes is essential for VAR2CSA vaccine development. Attempts to determine the crystallographic structures of VAR2CSA or its domains have not been successful yet. In this study, we propose 3D models for each of the VAR2CSA DBL domains and we show that regions in the fold of VAR2CSA inter-domain 2 and a PfEMP1 CIDR domain seem to be homologous to the EBA-175 and Pkα-DBL fold. This suggests that ID2 could be a functional domain. We also identify regions of VAR2CSA present on the surface of native VAR2CSA by comparing reactivity of plasma containing anti-VAR2CSA antibodies in peptide array experiments before and after incubation with native VAR2CSA. By this method we identify conserved VAR2CSA regions targeted by antibodies that react with the native molecule expressed on infected erythrocytes. By mapping the data onto the DBL models we present evidence suggesting that the S1+S2 DBL sub-domains are generally surface-exposed in most domains, whereas the S3 sub-domains are less exposed in native VAR2CSA. These results comprise an important step towards understanding the structure of VAR2CSA on the surface of CSA-binding infected erythrocytes.

## Author Summary

Individuals living in areas with high Plasmodium falciparum transmission acquire immunity to malaria over time and adults have markedly reduced risk of getting severe disease. However, pregnant women constitute an important exception, and they become more susceptible to malaria during pregnancy. This so called pregnancy-associated malaria (PAM) has severe consequences for both mother and child, and a vaccine would save hundreds of thousands of lives each year. PAM is caused by P. falciparum–infected red blood cells that bind to receptors in the placenta. By binding to the placental tissue, the parasites avoid being filtered though the spleen where they would have been killed. The protein mediating this placental binding is a very large multidomain and variant protein named VAR2CSA. Using structural modeling of VAR2CSA and antibody reagents from women who have had PAM, we show that antibodies tend to bind in similar regions, on one side of the individual VAR2CSA domains. In addition, we show that highly conserved parts of this variant protein are accessible for antibodies. This finding correlates with epidemiological data showing that woman acquire immunity towards PAM relatively fast, and the identification of these epitopes is thus a major step towards a protective vaccine.

## Introduction

Adhesion of Plasmodium falciparum parasite-infected erythrocytes (IE) to the vascular bed is mediated by P. falciparum erythrocyte membrane protein 1 (PfEMP1), which interacts specifically with receptors on the vascular endothelium or placenta [1,2]. The adhesion mechanism is thought to be developed by the parasite to avoid filtering through the spleen, where erythrocytes infected with late stage asexual parasites are removed from the circulation [3]. Antibodies that target PfEMP1 and abrogate binding are believed to be important mediators of acquired malaria immunity (reviewed in [4]). Pregnancy-associated malaria (PAM) is caused by P. falciparum sequestering in the placenta by binding to chondroitin sulfate A (CSA), which is a type of glycosaminoglycan attached on the surface of syncytiotrophoblasts [5]. Women suffering from PAM develop antibodies which protect them and their offspring during subsequent pregnancies [6]. These protective antibodies are thought to recognize a relatively conserved antigen as plasma and parasites from pregnant women from different malaria endemic areas cross-react [7]. The PfEMP1 variant mediating placental binding was recently discovered and named VAR2CSA [2,8]. The extracellular part of VAR2CSA consists of six Duffy-binding-like (DBL) domains, a large inter-domain (ID2) and a C-terminal region predicted to be cytoplasmic. Most PfEMP1 molecules, but not VAR2CSA, contain two cysteine-rich interdomain regions (CIDR domains) [9,10]. Some CIDR domains bind to CD36 [11] and they have been described as degenerated DBL domains [12] despite a very low sequence homology between DBL and CIDR domains.

The invasion of erythrocytes and the subsequent adhesion of IE to vascular endothelium or placenta are key events in the asexual life cycle of P. falciparum and thus of major importance for the virulence of this parasite. Erythrocyte invasion is mediated by proteins belonging to the erythrocyte binding ligand family (EBL) and in P. falciparum the erythrocyte binding antigen (EBA)-175 is the best described EBL protein. EBA-175 has some similarity to VAR2CSA: Firstly, EBA-175 contains two DBL domains (called F1 and F2). Secondly, the EBA-175 DBL domains bind glycans on the sialylated glycophorin A on the erythrocyte surface [13]. The monomeric structure of EBA-175 has been determined by X-ray crystallography and the primary features of the two DBL domains were found to be α-helices and an anti-parallel β-hairpin [14]. EBA-175 also crystallized as a dimer, and the structure of this complex showed that the DBL domains of EBA-175 interacted in a reverse handshake orientation [14]. The simian malaria parasite, Plasmodium knowlesi invades erythrocytes through the host receptor “Duffy antigen receptor for chemokines” (DARC) [15]. This interaction is also mediated by a parasite-encoded DBL-containing protein, Pkα-DBL, and the crystal structure of Pkα-DBL has been shown to be very similar to PfEBA-175 despite extensive sequence variation [16]. Based on the structure of Pkα-DBL, the DBL domain could be divided into three sub-domains named S1–S3 which are connected by short linkers. Both the glycan binding site of PfEBA-175 and the DARC binding site of Pkα-DBL are predominantly located in S1 and S2 [14,16].

With the aim of making a vaccine that can reverse or inhibit parasite binding in the placenta, considerable effort has been put into defining the specific part/parts of VAR2CSA that bind to CSA (reviewed in [17]). The best way of determining this interaction would be to produce the extracellular part of VAR2CSA and co-crystallize this multidomain protein with CSA. However, it is very difficult to express such a large protein and previous attempts to crystallize even single VAR2CSA DBL domains have failed. Thus, novel methods are required to generate testable models and hypotheses on the overall 3D structure of PfEMP1 molecules. The DBL domains of PfEMP1 are often illustrated as “pearls on a string” and vaccine development strategies are focusing on the VAR2CSA DBL domains as single entities in a larger protein. We have recently published data showing a structural model of VAR2CSA DBL3X and mapped areas of DBL3X that are surface-exposed and reactive to naturally acquired antibodies on the native protein [18]. In this previous study, we showed that most variable regions of DBL3X are located in flexible loops or surface-exposed parts of the model. These findings were supported by an analysis of 106 VAR2CSA sequences which was published recently [19], and showed that polymorphic sites in general are situated in flexible loop regions or other surface exposed areas of VAR2CSA DBL structure models.

In this study, we modeled the remaining five 3D7 VAR2CSA DBL domains, the VAR2CSA inter-domain 2 (ID2) and a number of CIDR domains from different PfEMP1 molecules to get insight into the location of epitopes in the whole VAR2CSA molecule. The models indicate that DBL domains contain features that are structurally conserved. Furthermore it appears that there is homology between the ID2, CIDR and part of the resolved structures of the DBL fold. By absorbing antibodies on native VAR2CSA on the surface of IE and comparing antibody reactivity on a VAR2CSA peptide array before and after absorption, we define areas of the VAR2CSA molecule which may be accessible to antibodies in the native protein. For all domains we find that relatively invariant parts are recognized and surface-exposed in the native VAR2CSA. The surface-exposed epitopes on the six VAR2CSA domains are largely found within S1 and S2, whereas S3 appears to be hidden in the complete VAR2CSA structure. This finding is interesting because it leads to the first suggestions about the overall structure of VAR2CSA; based on these data we discuss the domain architecture of VAR2CSA and suggest a model where the protein is surface-exposed as a globular or multimerized structured protein stabilized by long α-helices in the S3 region.

## Results/Discussion

### Modeling of the VAR2CSA DBL Domains

**Figure 1:**
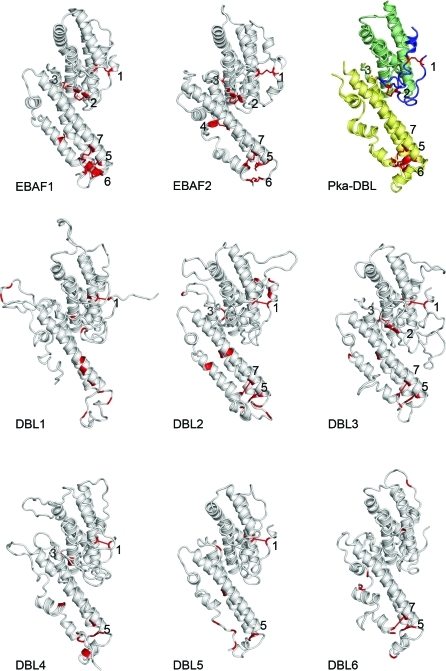
Structure Models of the VAR2CSA DBL Domains The experimental structures of the template EBA-175 and Pkα-DBL domains are shown in the first row, and DBL models are shown in the middle and last rows. Sub-domains 1–3 of Pkα-DBL (Pka-DBL) are colored in blue, green, and yellow, respectively. Cysteines in all domains are highlighted in red. Disulfide bonds in templates are numbered according to occurrence in the sequences and corresponding cysteine pairs in the models are numbered with respect to the template numbering.

**Figure 2:**
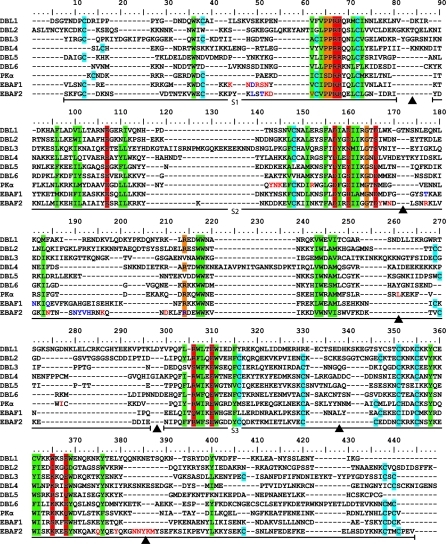
A Structural Alignment of the Template Structures EBA-175 F1 and F2 and Pkα-DBL and the Six VAR2CSA DBL Models Vertical color bars denote positions of stabilizing residues in template structures. Blue bars denote cysteines forming disulfide bonds, green bars denote buried hydrophobic residues, orange bars denote helix capping residues, and red bars denote buried polar/charged residues. The location of sub-domains S1–S3 are marked by horizontal black bars, and regions of missing structural information in the template structures are marked by black arrowheads. Residues involved in GAG binding of EBA-175 DBL domains or DARC receptor binding of Pkα-DBL are marked with red letters. EBA-175 residues involved in dimerization are marked in blue letters.

The 3-D structures of the 3D7 VAR2CSA DBL domains (Figure 1) were modeled using the HHpred server [20] developed for low-homology modeling. HHpred template searches confirmed that the determined structures of the P. falciparum EBA-175 DBL domains F1 and F2 and the P. knowlesi DBL domain Pkα-DBL [14,16] could be used as templates for modeling (HHpred probability scores were all 100%), although the sequence identity between the VAR2CSA DBL domains and templates was between 16–20% (see modeling details in Table S1). The determined structures of the two EBA-175 DBL domains each contain a region where structural information is missing and the structure of Pkα-DBL has four such regions (Table S1 and Figure 2). In crystallographic experiments, the local occurrence of missing structural information indicates regions of flexibility and loosely defined secondary structure. For Pkα-DBL, the regions of missing structural information were used to divide the DBL fold into the subdomains S1–S3 [14] and we adapted a similar classification for the VAR2CSA DBL domains (Figures 1 and 2). VAR2CSA residues corresponding to regions of missing structural information in the templates were modeled as insertions relative to the template structure. The structure of such inserted regions is difficult to predict correctly [21]. Secondary structure predictions using the PSIPRED method [22] is part of the HHpred modeling protocol. The prediction results are divided into helix, strand and coil, where the coil class consists of secondary structure types mostly found in loops. Interestingly, the template regions of missing structural information aligned with VAR2CSA DBL sequences predicted to have coil secondary structure. In general, the VAR2CSA sequence variation is high within these regions (Figure 2). Taken together, this suggests that most of the variable regions in all VAR2CSA DBL domains form a variety of flexible loops with different conformations. These results support the findings reported by Bockhorst et al., who recently reported a similar tendency in structural models of VAR2CSA DBL2X and DBL3X [19]. From a structural perspective these findings makes biological sense because the overall DBL fold could be preserved while surface-exposed parts and loop areas which are possibly less important for stabilization of the fold, would have more freedom to mutate.

### Evaluation of the VAR2CSA DBL Structure Models

The quality of a structure model obviously has a pronounced effect on the information that can be deduced from the model. We used the automated structure analysis tool ANOLEA [23] for evaluation, and Z-scores ranging from 5.00 to 9.61 with 52%–68% high-energy residues were obtained. The results indicated that the quality was lowest in the loop regions. Analysis using Verify3d [24] resulted in a similar conclusion (data not shown). Since these results did not convince us that the models were correct, we decided to further investigate the quality of the models by inspecting them for conserved residues stabilizing the determined structures of EBA-175 and Pkα-DBL domains.

Structural alignment of the EBA-175 and Pkα-DBL domains identified a number of positions, which can be assumed to be important for the stabilization of the DBL fold (Table S2). The positions of these residues were distributed throughout the domains in blocks. We then made a multiple structural alignment including the six VAR2CSA DBL models and the EBA-175 and Pkα-DBL structure to identify VAR2CSA residues at the positions corresponding to the positions identified as conserved and stabilizing EBA-175 and Pkα-DBL (Figure 2). This analysis showed that a high number of hydrophobic positions forming a hydrophobic core in the DBL structure were conserved in the models, together with positions of helix capping and positions of interacting buried polar residues (Table S2 and Figure 2). This conservation of stabilizing positions indicates that the alignments of the model sequences to the template sequences are correct in regions surrounding these positions. Additionally, we found that most template-stabilizing positions are located in semi-conserved blocks reported in an analysis of 106 VAR2CSA sequences [19] (data not shown), which suggests that these stabilizing positions in the templates are conserved in VAR2CSA to stabilize the folding of the VAR2CSA DBL domains in general. The structural alignment and sequence alignments used by HHpred for modeling were analyzed for conservation of cysteines forming disulfide bonds in the template DBL structures. The models of the VAR2CSA DBL domains all contain conserved cysteine positions likely to form disulfide bonds (Figures 1 and 2). The disulfide bonds were numbered according to the occurrence of the cysteines in the sequence. Cysteines of disulfide bond 1 are conserved in all models except DBL6. Likewise, the cysteines of disulfide bond 5 are conserved in all models except DBL1.

A number of cysteines in the models are in close proximity to a disulfide bond-forming template cysteine. This suggests that the local alignments used for the modeling are sub-optimal or that alternative disulfide bonds are formed in the VAR2CSA DBL domains.

An interesting example is the disulfide bond 2 (Figure 1, number 2 and Figure 2, positions 38 and 64). The disulfide bond is proximal to a region containing glycan-binding amino acids in the EBA-175 F1 and F2 domains (Figure 2, positions 44, 48, and 50–53) and it may play a role for the function of these domains. Among the VAR2CSA DBL domains, only DBL3 has both cysteines conserved. None of the other VAR2CSA DBL sequences have two cysteines in the proximity, and it is thus unlikely that the apparent variation stems from incorrect alignments. The lack of the disulfide bonds in some regions of the VAR2CSA DBL domains may suggest higher flexibility and a more dynamic structure than in DBL domains stabilized by a higher number of disulfide bonds.

The analysis of different types of stabilizing characteristics shows that these to a large extent are conserved between the template and the VAR2CSA models. Since our aim was to map experimental data onto the DBL models, rather than to analyze the structural conformations in detail, we concluded that the models were of sufficient quality for mapping of these data.

### Modeling of VAR2CSA Inter-Domain 2

**Figure 3:**
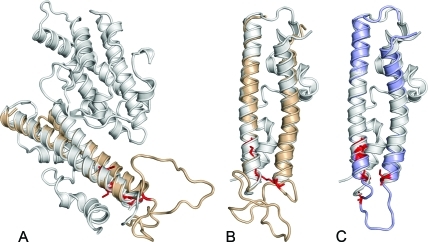
Modeled Regions of ID2 and CIDR Domains The S3 domain of the template EBA-175 F2 is shown in white. Cysteines in the models are highlighted in red. (A) The model of the VAR2CSA ID2 domain superimposed in yellow on the EBA-175 F2. (B) Similar to (A) except showing only the S3 domain. (C) Model of a CIDR domain.

The extracellular part of VAR2CSA consists of six DBL domains and a sequence stretch consisting of 337 amino acids named inter-domain 2 (ID2) [9]. This part of the molecule has attracted little attention and has been viewed as an inter-domain spacer sequence. We analyzed the ID2 sequence (PFL0030c positions 879-1216) for homology to other proteins using the HHpred search and alignment tool. Interestingly, the EBA-175 and Pkα-DBL domains were all identified as homologous to the ID2 domain with very high HHpred probability scores (probabilities between 98.3% to 99.9%). The similarity was pronounced in a region of 100 residues (PFL0030c positions 1017–1116), which aligned to the first two α-helices in S3 with a sequence identity of 19.8%. Using the EBA-175 F2 DBL domain as template, we modeled the structure of ID2 positions 1017–1116 (Figure 3A and 3B). The secondary structure of the whole ID2 domain was then predicted using PSIPRED (data not shown). The topology of predicted secondary structure elements in the ID2 domain suggested that the C-terminal part of ID2 has a similar fold to DBL S3, but did not support the notion that the N-terminal part of ID2 has a fold similar to DBL S1 and S2.

In most PfEMP1 molecules the first DBL domains are separated by a cysteine-rich inter-domain region (CIDR1) on which there is no structural data available. Using the HHpred server homology between CIDRs and EBA-175 was identified. To identify the significance of our results, and to make a more general analysis, we used a number of CIDR domains for the analysis. Since VAR2CSA ID2 and CIDR1 are placed either after the first or the second DBL domain and can be divided into sub-groups, we examined CIDR1 sequences representative for the three subgroups CIDR-alpha, beta and gamma. Similarly to the ID2 domain, the homology was detected in the first two helices of the DBL S3 and a structure model was made using the EBA-175 F2 DBL as template structure (Figure 3B). The homologous region was part of the CIDR M2 region defined by Smith et al. [10]. Similarly to our results of ID2, an analysis of topology in the predicted secondary structure suggested that C-terminal structures of CIDR domains are similar to the known structures of DBL S3 domains, but that the structure of the N-terminal may vary from DBL domains. These predictions suggest that like ID2, the C-terminal part of CIDR1 seems to form a structure similar to that of the DBL S3.

### Mapping Surface-Expressed Continuous Epitopes on VAR2CSA

In rational PAM vaccine design, it is important to establish which parts of native VAR2CSA are accessible to antibodies acquired by women who have developed immunity to pregnancy-associated malaria. Additionally, the cross-reactivity of these antibodies is an important issue to investigate because of the variability in VAR2CSA sequences. The latter has been addressed in several studies which have been investigating the cross-reactivity of naturally acquired human antibodies between different P. falciparum lines. Both cross-reactivity [25,26] and isolate-specific recognition of antibodies have been reported [26,27]. These studies were based on antibody reactivity to IE and the specificity as well as the target of the cross-reactive and isolate-specific antibodies are thus not known. In our study, we have instead used VAR2CSA peptide arrays measuring binding to shorter peptides.

Anti-VAR2CSA IgG from pools of plasma was absorbed using VAR2CSA-expressing IE and the antibody reactivity of the pools in a VAR2CSA peptide array compared before and after absorption [18]. Measuring the antibody reactivity in the peptide assays allowed qualitatively mapping of surface-exposed regions of the VAR2CSA DBL domains. Plasma samples from 180 Tanzanian women, sampled at the time of delivery, were tested for reactivity towards CSA binding parasites (3D7CSA and FCR3CSA) in flow cytometry and in ELISA towards recombinant VAR2CSA protein. Two pools with high levels of antibodies towards IE and high levels of anti-VARCSA IgG were made using plasma from 32 and 10 Tanzanian pregnant women, respectively. One of the plasma pools (Human plasma pool 1) was depleted using erythrocytes infected with 3D7CSA parasites. From the other pool (Human plasma pool 2), two depleted plasma pools were generated by depleting one part with erythrocytes infected with 3D7CSA, and depleting the other part on erythrocytes infected with the FCR3CSA parasite line. As a control, we depleted a pool containing anti-VAR2CSA IgG (Human plasma pool 3) on a 3D7 parasite expressing a non-VAR2CSA PfEMP1 variant (VAR4). The antibody assays were performed on an array containing 442 overlapping 31mer peptides corresponding to the extracellular part of VAR2CSA based on the sequence of 3D7.

Before absorption, we observed a number of intense peaks for most of the domains (Figures S1–S6). The intensity varied between domains, for instance, the peptide reactivity in DBL1 resulted in highly intense peaks, whereas the peptide activity of DBL2 resulted in less intense peaks. The antibody reactivity towards the majority of peptides was not affected by the depletion; nevertheless depletion on native VAR2CSA consistently removed antibody reactivity against some peptides in all domains (Figures S1–S6). In general depletion on the non-CSA binding parasite did not reduce anti-VAR2CSA reactivity in the peptide array, but a small reduction of peptide-specific reactivity was detected in regions for DBL2 and DBL4. For DBL4 there was an overlap in the depletion on the non-CSA binding parasite and the CSA-binding parasite, indicating that some of the surface-exposed parts identified in DBL4 could be due to non-specific absorption.

In addition to the human plasma pool, a pool of sera from six rabbits each immunized with a different VAR2CSA DBL domain was absorbed and tested. The pattern of reactivity in the peptide array with this pool before absorption was slightly different from the reactivity obtained with the human plasma pools and this difference was also reflected in the absorption experiments.

Taken together these results indicated that all domains including the N-terminal segment contained continuous peptide sequences accessible to antibodies when the VAR2CSA protein was expressed on the surface of CSA-binding IE. It was difficult however, to detect a pattern for this reactivity between the domains when depicting the reactivity on a string of residues. We therefore went on to visualize the reactivity on the DBL models.

### 3-D Aspects of Surface-Exposed Epitopes in VAR2CSA DBL Domains

**Figure 4:**
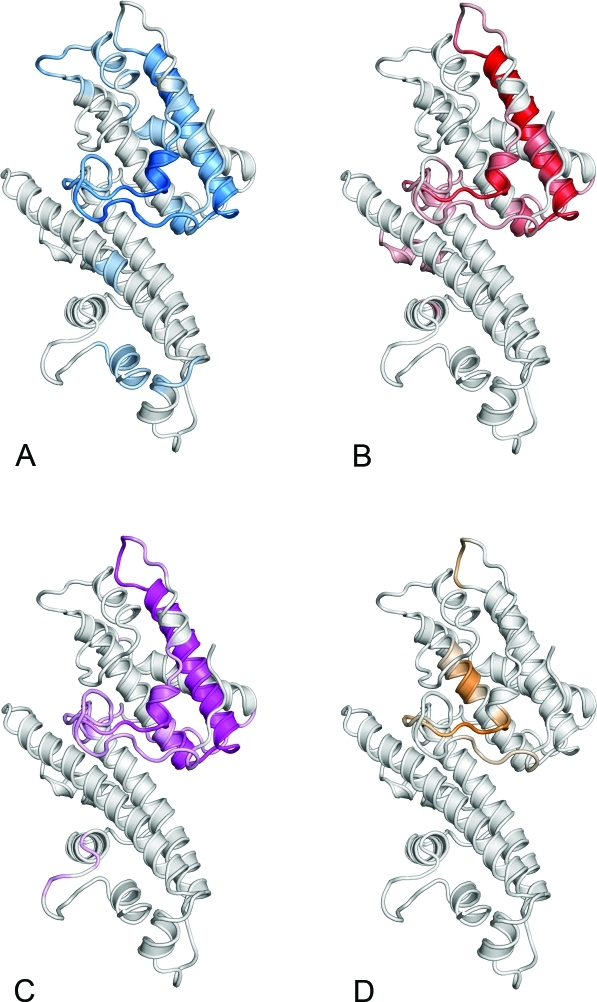
Mapping of Surface-Exposed Antibody-Reactive Regions onto the Model of the DBL6 Domain The color intensity denotes the level of antibody depletion when VAR2CSA containing plasma is incubated with parasites expressing native VAR2CSA. The following plasma and parasite combinations were used for the depletion experiments. (A) Human plasma pool 1 depleted with 3D7 parasites. (B) Human plasma pool 2 depleted with 3D7 parasites. (C) Human plasma pool 2 depleted with FCR3 parasites. (D) Rabbit plasma pool depleted with 3D7 parasites.

**Figure 5:**
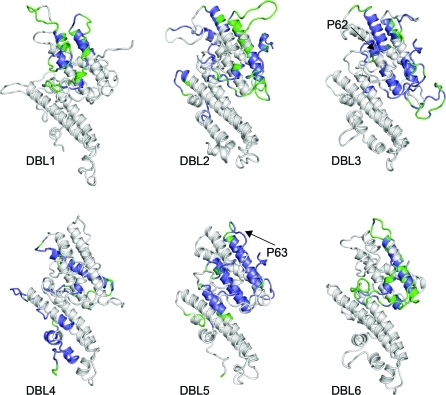
Areas Predicted to be Targeted by Surface-Reactive Antibodies on the Six VAR2CSA DBL Domains The highlighted regions were defined on the basis of the results of several experiments where anti-VAR2CSA antibody containing plasma was incubated with parasites expressing native VAR2CSA. Residues with consensus DVs > 1 which are conserved in sequences of VAR2CSA are highlighted in blue, and variable residues with consensus DVs>1 are highlighted in green. See text for details. The positions of DBL3 and DBL5 peptides (P62 and P63 respectively) used for affinity purifying IgG (see Figure 8) are marked with black arrows.

To facilitate the mapping of depleted regions from the peptide array data we calculated depletion values (DV) by subtracting the depleted reactivity from the non-depleted reactivity. DV were calculated for each of the four depletion experiments (human pool 1 versus 3D7 or FCR3, human pool 2 versus 3D7, rabbit pool versus 3D7) and mapped onto the six structural DBL models (Figures S7–S11). Figure 4 shows the results for DBL6 and it is apparent that the depletion experiments using the two human plasma pools identified essentially the same regions as target for surface reactive antibodies (Figure 4A versus 4B). The results obtained in the absorption experiment using FCR3-infected erythrocytes gave essentially the same results as the experiment using 3D7-infected cells (Figure 4B versus 4C). Since the peptide array was based on the 3D7 sequence this indicates that the surface-exposed epitopes on the 3D7 and FCR3 versions of VAR2CSA are cross-reactive or target-conserved epitopes. The absorption experiment using the rabbit plasma pool only showed depletion of antibodies targeting peptides residing in S1 and S2 (Figure 4D). There was no indication of depletion of antibodies targeting S3 in any of the experiments. To facilitate a comparison between the DBL domains, and to reduce the risk of false positive DV, we created consensus DVs for each domain by calculating a sum of normalized DV from each of the four absorption experiments and scoring the residue as positive if the value was above a fixed threshold (Figure 5). Overall there was agreement between the models based on the individual absorption experiments and the consensus DV (Figure 4A and 4B versus Figure 5, DBL6). The results for DBL3 were also in agreement with the surface-exposed epitopes identified previously [18]. When evaluating the consensus models it should be kept in mind that surface-exposed VAR2CSA regions can only be detected if the plasma pool contains antibodies against these regions and if the antibodies can be detected in the peptide array assay. Thus, regions which are not targeted by antibodies, or regions targeted by antibodies that cannot be detected in the peptide array experiment (because they either target non-linear sequences or polymorphic sequences not represented in 3D7 VAR2CSA,) will not be scored as surface reactive. As a consequence this method will only map a certain proportion of epitopes. Likewise, residues buried in the native molecule but residing close to a region representing a surface-exposed epitope on the peptides will score as positive.

When comparing the consensus models for the six DBL domains, it was evident that the pattern of reactivity was comparable in DBL domains 1, 2, 3, 5, and 6, whereas the pattern in DBL4 was unique (Figure 5). For the former domains the targets of surface-reactive antibodies were mainly located in S1 and S2, whereas little reactivity was found in S3, which is located on the lower left side of the models in Figure 5. In S1 and S2 both loops and α-helices were targets of surface reactive antibodies. The loop between S1 and S2 (Figure 2 positions 81–87 and appearing most prominently in the upper left corner of the DBL2 model on Figure 5) is flexible in the Pkα-DBL structure and we observe a high sequence variation between the DBL domains in the region, suggesting that the corresponding loops in the VAR2CSA DBL domains are flexible, and reinforcing the possibility that VAR2CSA domains can be divided into sub-domains. In DBL domains 2, 3, 5, and 6, a loop in S2 (appearing most prominently in the lower right corner of DBL3 on Figure 5) was also targeted by surface reactive antibodies. A loop region in S1 (Figure 2, positions 44–52, appearing most prominently in the center of DBL6 on Figure 5) was recognized in DBL domains 1, 2, 3, and 6. Interestingly, the corresponding regions in EBA-175 contain glycan-binding residues. The S2 α-helix appearing in the upper right on all models was recognized in all domains but DBL4, whereas some of the other α-helices in S1 and S2 were recognized to a varying degree.

**Figure 6:**
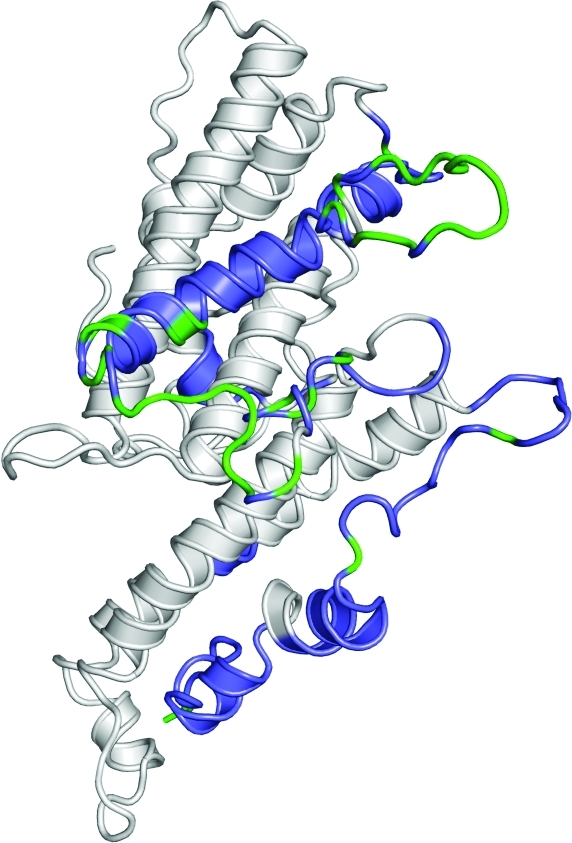
VAR2CSA DBL4 Regions Predicted to be Targeted by Surface-Reactive Antibodies The plot is similar to Figure 4, except viewed from the reverse side.

The regions of DBL4 targeted by surface-reactive antibodies differed markedly from the other domains. Relatively more reactivity was detected against S3 and the reactivity against S2 was mainly against regions on opposite side of the domain compared to the other domains (Figures 5 and 6). The possibility that DBL4 is positioned different from the other domains in the quaternary VAR2CSA structure is in agreement with the finding that the DBL4-specific rabbit antibodies are not reacting with the native VAR2CSA on IE ([28] and unpublished data). The different pattern of DBL4 recognition could also be explained by the fact that some antibodies targeting DBL4 peptides were non-specifically absorbed on IE (Figure S4, lower panel).

There is no evidence that S3 is undergoing less diversifying selection, which might be expected considering the present experimental data. To address whether epitopes in S3 in particular are conformationally arranged compared to S1+S2 and thus showing a bias in the results, we affinity purified rabbit antibodies on monomeric recombinant DBL2 and assessed these antibodies on the peptide array. We found that epitopes in both S2 and S3 could be detected by the peptide array analysis (data not shown), indicating that the system was functioning for epitopes in S3 as well.

Using a multiple sequence alignment of seven full-length VAR2CSA sequences, residues were classified as conserved if they were all identical and as polymorphic if any of the sequences showed variation in the particular position. It is apparent from Figures 5 and 6 that all domains contained both conserved and polymorphic regions targeted by surface reactive antibodies, but the conserved regions were most prominent in DBL3 and DBL5. This is in agreement with data showing that antibodies raised against recombinant proteins representing DBL3 and DBL5 are more likely to cross-react with heterologous parasites, than antibodies raised against the other domains [28]. Furthermore, these findings also support data showing that antibodies raised against one DBL3 or DBL5 variant are highly cross-reactive with a large panel of placental isolates (Magistrado et al., submitted). Finally, the data is also in agreement with the reactivity of human monoclonal antibodies produced by immortalized B cells from malaria-exposed pregnant women which are directed predominantly against these two domains [29].

### Conserved Surface-Exposed VAR2CSA Epitopes

**Figure 7:**
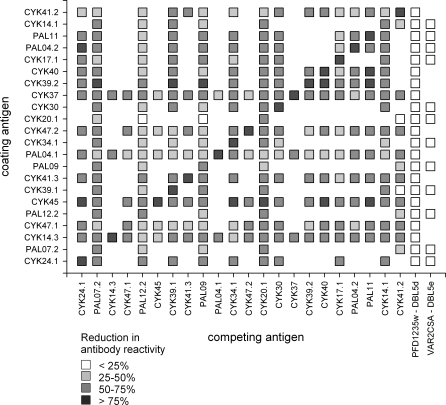
A Matrix Showing the Level of Cross-Reactive Antibody Targets between 22 Placental VAR2CSA DBL3 Variants Produced in *Baculovirus*-Infected Insect Cells Cross-reactivity was determined by competition ELISA using a Tanzanian female plasma pool with a high titer of VAR2CSA-specific antibodies. Responses with the same protein as the competing and coating antigen are shown in the diagonal. Blanks with no square are protein combinations not tested. The identity of the various DBL3 proteins corresponds to the nomenclature used in the published multiple sequence alignment [18]. Control proteins included a DBL5 VAR2CSA protein and a non-VAR2CSA DBL domain (PFD1235w).

To further investigate the presence of conserved immunogenic epitopes in VAR2CSA domains, we performed competition ELISA using a large panel of recombinant VAR2CSA DBL3 domains derived from placental isolates [18]. One variant of VAR2CSA DBL3 was coated in ELISA plates and the antibody reactivity of a high-titered VAR2CSA plasma pool was compared before and after pre-incubation with a competing VAR2CSA DBL3 variant. By this method it was possible to quantify the relative level of variant-specific IgG towards individual DBL3 variants. As positive and negative controls we incubated the plasma pool with homologous VAR2CSA DBL domain or VAR2CSA DBL5 domain as competing antigen (Figure 7). Pre-incubation with non-homologous DBL3 domain reduced the reactivity by 25%–100%. No competition/absorption was seen with the control proteins. These data strongly suggest that the insect-cell produced DBL3 domains share common and cross-reactive motifs.

**Figure 8:**
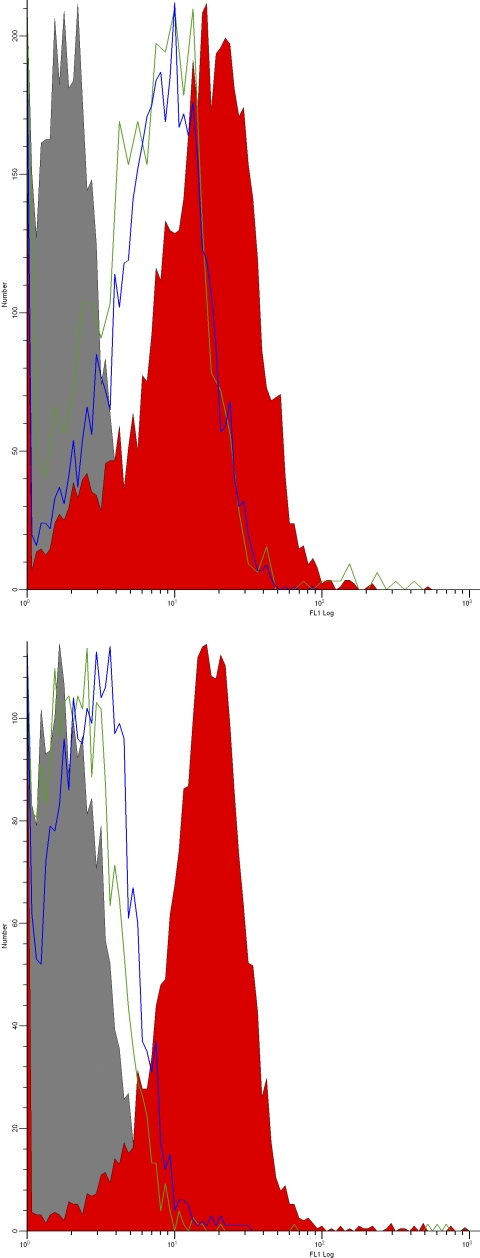
Surface Reactivity (Fitch FL1) of Rabbit Antibodies Targeting Conserved VAR2CSA Regions Rabbit antibodies were affinity purified on peptides (P62 and P63; see Figure 5 for details) and tested for reactivity with IE expressing VAR2CSA. The parasite lines FCR3CSA (A) and 3D7CSA (B) IE were used. The following antibodies were used: grey, IgG control; red, VAR2CSA-specific rabbit pool; blue, P63 (DBL5)-specific antibodies; green, P62 (DBL3)-specific antibodies. For FCR3, the number of cells staining positive with the VAR2CSA pool, P63 and P62 were 97%, 56%, and 48%, respectively. For 3D7, 85%, 7%, and 6% of the cells were positive.

To determine whether some of these conserved linear regions were exposed on the surface of the native parasite protein, we synthesized peptides corresponding to two regions in DBL3 and DBL5 suggested by the peptide array data to be surface-exposed (Figure 5). The first peptide P62, corresponded to aa position 1350–1370 in DBL3. This region is highly conserved having only one variant aa (based on alignment of 43 VAR2CSA DBL3 sequences). The second peptide, P63, corresponds to aa 2045–2061 in DBL5 and is also relatively conserved having 2 variant aminoacids out of 17 (based on alignment of 15 VAR2CSA DBL5 sequences). P62 and P63 were tested in ELISA for reactivity with Tanzanian male and female plasma and both showed significantly higher reactivity with female plasma as compared to male plasma (Mann-Whitney rank sum test, *p* < 0.05; data not shown). The two peptides were screened in ELISA for reactivity against plasma from rabbits immunized with recombinant DBL3 or DBL5 protein and peptide-reactive rabbit sera was used to affinity purify antibodies on the peptides to create peptide-specific IgG reagents. The affinity-purified antibodies were subsequently assayed in flow cytometry for reactivity with native VAR2CSA expressed on erythrocytes infected with CSA-binding 3D7 or FCR3 strain (Figure 8). Both the DBL3 and DBL5 peptide purified antibodies reacted strongly with FCR3CSA strain and to lesser extend with the 3D7CSA parasite. The DBL5 peptide antibodies were affinity-purified from a rabbit immunized with a FCR3 DBL5 domain, and the difference of two amino acids in the peptide sequence could explain the lower reactivity with 3D7CSA compared with FCR3CSA. The DBL3 peptide-specific antibodies were affinity purified from a rabbit immunized with a placental variant of DBL3 protein and there is only one amino acid differing between this placental sequence and the FCR3 and 3D7 sequences. It was intriguing to find that the affinity-purified antibodies recognized the parasites to varying degree. One reason for this could be a difference in levels of VAR2CSA protein expressed on 3D7 and FCR3, or the observed difference could be accounted for by the single polymorphisms in the peptide sequences. A third explanation could be that polymorphic flexible loop regions, flanking the conserved surface exposed parts, influence the accessibility of the surface-exposed regions to varying degrees between 3D7 and FCR3.

Conserved surface-exposed epitopes appear to be attractive vaccine targets. However, protective immunity is acquired through successive pregnancies [6,30], and is a function of transmission intensity [31]. Naturally acquired protection could therefore depend on the ability to recognize several polymorphic VAR2CSA variants. This is consistent with the finding that some targets of naturally acquired antibodies are under diversifying selection [18]. The levels of antibodies against pregnancy-associated parasite-encoded antigens on the surface of the erythrocyte increase with the number of pregnancies, and are correlated to the adhesion inhibitory capacity [7,32]. Therefore, our identification of antibody binding parts in more conserved regions of VAR2CSA may seem like a paradox. However, there could be several explanations for this finding: Firstly, the identified regions may have importance for the function of VAR2CSA, and this could lead to conservation. Second, the conserved regions may be less immuno-dominant than other more variable regions of VAR2CSA. This would make the development of antibodies directed against these regions less frequent, and result in a delay in the development of immunity. Finally, whole antibody binding surfaces may be composed of both conserved and variable regions, where the variance of a few residues would be enough to disrupt the binding. Most antibody binding epitopes of globular proteins have been estimated to be discontinuous in nature [33], and additionally it has been shown that most discontinuous epitopes are comprised by 14–19 amino acids, including a linear segment of 4–7 amino acids [34]. Therefore, the complete binding surface of antibodies recognizing P62 or P63 could very likely be discontinuous, and comprised by both polymorphic and more conserved regions.

We have identified conserved VAR2CSA regions targeted by antibodies recognizing the surface of IE. Furthermore we have been able to induce rabbit antibodies against these regions by immunization with recombinant DBL domains. This is a promising finding for vaccine development and it will be important to establish whether antibodies against conserved surface-exposed VAR2CSA regions can inhibit the binding of parasites to CSA.

### VAR2CSA, a Globular Protein?

So far, little is known about the overall structure of VAR2CSA or any other PfEMP1. It has been suggested that the PfEMP1 protein architecture is comprised by a compact semi-conserved head structure which largely defines the binding affinity and a number of variable C terminal domains [12]. Previous data have indicated that the general DBL fold is relatively conserved [14,16,18,35] and that DBL domains can interact with each other as building blocks to form binding sites [14]. The data presented here indicate that DBL S3 is less surface-exposed than S1 and S2. Sub-domain 3 contains two long α-helices which are conserved in the template structures. A number of multimeric protein complexes have been reported to be stabilized by interactions between long α-helices; a well-studied example is the trimer of influenza virus hemagglutinin [36]. The data presented here does not lend support to the idea of a compact conserved head structure in VAR2CSA. Rather it is tempting to propose models of VAR2CSA where α-helices of different DBL domains interact to bury a considerable part of S3 in the interface between the domains. The interaction could be formed between DBL domains of a single VAR2CSA molecule to form a more globular shape of VAR2CSA. Another possibility is that several VAR2CSA molecules form dimers or multimers with S3 buried in the middle. These models do not exclude the possibility that the flexible loop regions, also present in S3, protrude from the compact core structure of the protein [29], however we were not able to measure antibodies to these regions using the peptide array. The hypothesis that VAR2CSA is present as a globular protein opens up for the possibility that the CSA binding site is comprised of regions from different DBL domains, like the glycan binding sites of EBA-175 [14]. Further exploration of the quaternary structure of VAR2CSA is therefore of the utmost importance.

## Materials and Methods

### Modeling the structure of the VAR2CSA domains.

Structures of the CIDR domains and the 3D7 VAR2CSA DBL and ID2 domains were modeled using the HHpred server with default settings [20]. The HHpred method is based on comparisons and alignments of hidden Markov models (HMMs), which include gaps and insertion probabilities. The modeling of the DBL3 domain was done as described previously [18]. VAR2CSA domains were modeled separately by splitting the PFL0030c sequence into separate domains (DBL1 aa 57–400, DBL2 aa 531–879, DBL4 aa 1575–1911, DBL5 aa 1999–2283, DBL6 aa 2340–2633, ID2 aa 879-1216). All HMM databases available in web-server were used for template structure search, including the Protein Data Bank (PDB). For all the VAR2CSA DBL domains described, the structures of the EBA-175 DBL domains [14] and the Pkα-DBL domain [16] had HHpred probability scores significantly higher than other structures detected. The DBL structure with the highest sequence and secondary structure alignment scores were chosen as template for each domain. Template alignments proposed by the HHpred method were used to generate 3D models by using a HHpred server toolkit protocol for MODELLER [37]. Models were evaluated using Verify3d [24] and ANOLEA [23], available in the HHpred server toolkit. Superimpositions of EBA-175 F1, F2, Pkα-DBL and VAR2CSA domains were made using the MAMMOTH multi-alignment server [38]. NACCESS version 2.1.1 [39] was used for analysis of relative accessible surface areas (RSAs) in the template structures. Residues with RSA < 30% were considered buried. Separate secondary structure predictions of CIDR and ID2 were made using the PSIPRED [22]. All structural visualizations were produced using PyMol [40].

### Selection and depletion of plasma samples on parasites.

Plasma samples sampled at the time of delivery from Tanzanian woman were tested in flow cytometry for the presence of antibodies against CSA binding parasites. Positive samples were selected for further analysis in ELISA; plasma levels of DBL1-DBL6 VAR2CSA specific IgG were measured in standard ELISA assays [41]. Plasma samples positive against more than two domains were used to make two pools of plasma samples. No single plasma sample was used in both pools. Plasma originated from women with different parities ranging from one to seven. The control human plasma pool 3 was a hyperimmune pool made from 7 Tanzanian women and 3 male. For testing the recognition pattern of the two synthetic peptides P62 and P63 we used a panel of Tanzanian female plasma which was selected on the basis of having high levels of antibodies towards CSA binding parasites and as controls we used Tanzanian male plasma. In addition we tested this panel of male and female plasma for reactivity to GLURP protein to ensure that we were not merely measuring different levels of malaria exposure. Rabbits were immunized with DBL1–DBL6 and ID2 recombinant protein as described [42] and the seven serum samples were pooled to create the VAR2CSA rabbit pool.

3D7 and FCR3 parasites were panned on BeWo cells to create CSA adhering parasite lines. Using real time PCR and flow cytometry with VAR2CSA specific reagents as well as plasma from Tanzania, we verified that the parasites were gender-specifically recognized and expressing high levels of VAR2CSA on the surface of the infected erythrocyte [43]. Ethical clearance for collection of plasma samples was given by the Tanzanian health authorities.

For the parasite IgG depletion 40 μl of the plasma pool were incubated with 2.0 × 10^8^ MACS purified intact late stage trophozoite- and schizont-IE for 20 min at 4 °C. Hereafter, the cells were centrifuged at 800*g* for 8 min, and the supernatant used to suspend a new pellet of 2.0 × 10^8^ infected erythrocytes. This procedure was repeated four times. Depletion on parasites was performed sequentially until iRBC reactivity could not be reduced further as determined by flow cytometry (see Figure S12).

### Analysis of Pepscan data.

A Pepscan array containing 442 31mer peptides corresponding to the extracellular part of 3D7 VAR2CSA was used for antibody binding studies. The sequences of the peptides had an overlap of six residues and the purity of the peptides was expected to be 70% or higher. All Pepscan data were analyzed for short motifs to derive the activity of single residues as described previously [18]. The use of the motif analysis ensured that reactivities of single residues were derived from several peptides in order to take effects of possible impurities into account.

As part of the motif analysis, the data from each individual experiment were normalized based on the average activity measured for the total array. This was done to avoid experimental errors caused by inter-assay variation. However, this procedure caused peaks remaining after depletion to gain intensity, because the depletion of other peaks lowered the general average of the data. Care was taken to avoid mis-interpretations of data caused by a gain in intensity upon depletion. For each of the four depletion studies, DVs were calculated by subtracting depleted Pepscan data points from non-depleted points. Peaks that have a diminished intensity after depletion lead to positive DVs. Therefore only positive DVs of each individual experiment were split into five equally sized intervals ranging from the highest to the lowest DVs and with increasing color intensity. The five intervals were then plotted on the models. For consensus DVs, the DVs for each depletion study on individual DBL domain were first normalized by subtracting the mean and dividing with the standard deviation. Subsequently, the consensus DVs was calculated by summing the normalized values for each position in the DBL domains. Consensus DVs > 1 were plotted on the models as this threshold ensured that only the peaks with the highest consensus DVs were included.

### Production of recombinant proteins and competition ELISA.

All VAR2CSA DBL3 constructs derived from placental isolates were cloned and sequenced as described elsewhere [18] and the DBL5δ domain of PFD1235w was cloned as described in [44]. The DBL5 domain of VAR2CSA was amplified from genomic FCR3 DNA with following primers: 5′ CC CCC GGG AGA TGT TTT GAT GAT CAG ACA and 3′ ATT TGC GGC CGC CAT TAC CTT TAT CAT ACT C and cloned into the pAcGP67-A vector as described for the DBL3 constructs. Recombinant *Baculovirus* particles were generated in Sf9 insect cells by co-transfecting with pAcGP67-A and linearized Bakpak6 *Baculovirus* DNA (BD Biosciences). Recombinant constructs were purified on a HIS-Select Nickel Affinity Gel (H8286, SIGMA) as secreted histidine-tagged proteins from the supernatant of virus-infected High-Five insect cells.

Prior to competition ELISA, all DBL3 constructs and the DBL5ɛ of VAR2CSA were tested in indirect ELISA for antibody reactivity by the female plasma pool (diluted 1:100) of 10 Tanzanian women (Korogwe, mixture of primi- and multigravidae, selected based on their high anti-VAR2CSA titers in flow cytometry) and OD490 values ranged from 1.3 to 3.2.

MaxiSorp microtiter plates (Nunc) were coated with antigen (1.5 μg/ml in PBS) overnight at 4 °C. The female plasma pool (diluted 1:100) was pre-absorbed with competing antigen (1 μg/ml) for 2 h at room temperature (RT). After incubating the plates with blocking buffer (PBS, 0.5 M NaCl, 1% Triton X-100, 1% BSA) for 1 h at RT, the pre-absorbed pool was added to the antigen-coated wells in triplicate and incubated for 1 h at RT. In addition to the pre-absorbed plasma pool (pP), a non-absorbed pool (P) was included for each coating antigen. Following washing of the plates four times with washing buffer (PBS, 0.5 M NaCl, 1% Triton X-100, pH 7.4), the secondary antibody (rabbit anti-human IgG HRP, P0214, Dako, Denmark) diluted 1:3000 in blocking buffer was added and incubated for 1 h at RT. Plates were again washed four times and antibody reactivity visualized by the addition of o-phenylenediamine substrate. Color reactions were stopped by the addition of 2.5 M H2SO4 and OD was measured at 490 nm. The percent reduction in antibody reactivity was calculated as follows: 100*[1-ODpP/ODP].

### Peptides and affinity purification of antibodies.

Peptide synthesis was done at Sigma Genosys at at X > 70% purity. Two conserved, surface-exposed regions were synthezised: P62-DBL3 YKNMILGTSVNIYEYIGKLQ residing at aa position 1350–1370 in the 3D7 sequence and P63-DBL5 RIVRGPANLRNLKEFKE residing at aa position 2045–2061.

Affinity purification of antibodies was done using HiTrap NHS-activated HP columns (GE Healthcare, http://www.gehealthcare.com/) according to the manufacturer's instructions. In brief, 1.5 mg of synthetic peptide (Sigma-Genosys) was dissolved in 0.2 M NaHCO_3_, 0.5 M NaCl (pH 8.3), and applied to the 1 ml column that had been equilibrated with 3 × 2 ml 4 °C 1 mM HCl. After coupling, the columns were washed alternating 0.5 M ethanolamine, 0.5 M NaCl (pH 8.3) and 0.1 M acetate, 0.5 M NaCl (pH 4), followed by a final wash with PBS (pH 7.4). Before affinity purification, the peptides were tested for reactivity against a panel of rabbit sera immunized with different recombinant DBL domains. A rabbit plasma sample reacting with one peptide was chosen to affinity purify antibodies specific for that peptide. Serum from a rabbit immunized with a placental DBL3 variant (accession number ABK91125) was used to affinity purify on P62 and serum from a rabbit immunized with DBL5 FCR3 was used to affinity purify antibodies on P63. Rabbits immunized with the 3D7 VAR2CSA DBL3 and DBL5 protein did not have antibodies against the peptides. 1 ml of rabbit plasma was diluted in PBS (1:2), filtered through a 0.45-μm filter and applied to the column at a flow rate of 0.5 ml / min. After washing the column in 5 ml PBS, affinity-bound antibodies were eluted in fractions with a total volume of 3.2 ml of 0.1 M glycine-HCl (pH 2.8) and neutralized in 1 M Hepes (pH 8).

The specificity of the purified antibodies was tested in ELISA against (1) the peptide used for affinity purification (2) the recombinant DBL domain used to immunize the rabbit that provided the plasma (3) another VAR2CSA domain.

## Supporting Information

Figure S1 Plots of Pepscan Analysis Results for the DBL1 Domain The top figure represents results from anti-VAR2CSA antibody human plasma pool 1 before and after depletion with infected parasites. The second figure from the top represents similar results obtained by using anti-VAR2CSA antibody human plasma pool 2, and the third figure from the top represents similar results obtained by using a rabbit anti-VAR2CSA antibody plasma pool. The bottom figure shows the control human plasma pool 3, which was depleted using a non-CSA binding parasite.
(68 KB EPS)

Figure S2 Plots of Pepscan Analysis Results for the DBL2 Domain The top figure represents results from anti-VAR2CSA antibody human plasma pool 1 before and after depletion with infected parasites. The second figure from the top represents similar results obtained by using anti-VAR2CSA antibody human plasma pool 2, and the third figure from the top represents similar results obtained by using a rabbit anti-VAR2CSA antibody plasma pool. The bottom figure shows the control human plasma pool 3, which was depleted using a non-CSA binding parasite.
(69 KB EPS)

Figure S3 Plots of Pepscan Analysis Results for the DBL3 Domain The top figure represents results from anti-VAR2CSA antibody human plasma pool 1 before and after depletion with infected parasites. The second figure from the top represents similar results obtained by using anti-VAR2CSA antibody human plasma pool 2, and the third figure from the top represents similar results obtained by using a rabbit anti-VAR2CSA antibody plasma pool. The bottom figure shows the control human plasma pool 3, which was depleted using a non-CSA binding parasite.
(69 KB EPS)

Figure S4 Plots of Pepscan Analysis Results for the DBL4 Domain The top figure represents results from anti-VAR2CSA antibody human plasma pool 1 before and after depletion with infected parasites. The second figure from the top represents similar results obtained by using anti-VAR2CSA antibody human plasma pool 2, and the third figure from the top represents similar results obtained by using a rabbit anti-VAR2CSA antibody plasma pool. The bottom figure shows the control human plasma pool 3, which was depleted using a non-CSA binding parasite.
(68 KB EPS)

Figure S5 Plots of Pepscan Analysis Results for the DBL5 Domain The top figure represents results from anti-VAR2CSA antibody human plasma pool 1 before and after depletion with infected parasites. The second figure from the top represents similar results obtained by using anti-VAR2CSA antibody human plasma pool 2, and the third figure from the top represents similar results obtained by using a rabbit anti-VAR2CSA antibody plasma pool. The bottom figure shows the control human plasma pool 3, which was depleted using a non-CSA binding parasite.
(60 KB EPS)

Figure S6 Plots of Pepscan Analysis Results for the DBL6 Domain The top figure represents results from anti-VAR2CSA antibody human plasma pool 1 before and after depletion with infected parasites. The second figure from the top represents similar results obtained by using anti-VAR2CSA antibody human plasma pool 2, and the third figure from the top represents similar results obtained by using a rabbit anti-VAR2CSA antibody plasma pool. The bottom figure shows the control human plasma pool 3, which was depleted using a non-CSA binding parasite.
(61 KB EPS)

Figure S7 Mapping Surface-Exposed Antibody-Reactive Regions on the Model of the DBL1 Domain The color intensity denotes the values of positive depletion values.
(A) Human plasma pool 1 depleted with 3D7 parasites.
(B) Human plasma pool 2 depleted with 3D7 parasites.
(C) Human plasma pool 2 depleted with FCR3 parasites.
(D) Rabbit serum pool depleted with 3D7 parasites.
(2.5 MB TIF)

Figure S8 Mapping Surface-Exposed Antibody-Reactive Regions on the Model of the DBL2 Domain The color intensity denotes the values of positive depletion values.
(A) Human plasma pool 1 depleted with 3D7 parasites.
(B) Human plasma pool 2 depleted with 3D7 parasites.
(C) Human plasma pool 2 depleted with FCR3 parasites.
(D) Rabbit serum pool depleted with 3D7 parasites.
(2.8 MB TIF)

Figure S9 Mapping Surface-Exposed Antibody-Reactive Regions on the Model of the DBL3 Domain The color intensity denotes the values of positive depletion values.
(A) Human plasma pool 1 depleted with 3D7 parasites.
(B) Human plasma pool 2 depleted with 3D7 parasites.
(C) Human plasma pool 2 depleted with FCR3 parasites.
(D) Rabbit serum pool depleted with 3D7 parasites.
(2.8 MB TIF)

Figure S10 Mapping Surface-Exposed Antibody-Reactive Regions on the Model of the DBL4 Domain The color intensity denotes the values of positive depletion values.
(A) Human plasma pool 1 depleted with 3D7 parasites.
(B) Human plasma pool 2 depleted with 3D7 parasites.
(C) Human plasma pool 2 depleted with FCR3 parasites.
(D) Rabbit serum pool depleted with 3D7 parasites.
(2.4 MB TIF)

Figure S11 Mapping Surface-Exposed Antibody-Reactive Regions on the Model of the DBL5 Domain The color intensity denotes the values of positive depletion values.
(A) Human plasma pool 1 depleted with 3D7 parasites.
(B) Human plasma pool 2 depleted with 3D7 parasites.
(C) Human plasma pool 2 depleted with FCR3 parasites.
(D) Rabbit serum pool depleted with 3D7 parasites.
(2.5 MB TIF)

Figure S12 Depletion of Female Plasma on Infected Erythrocytes A pool of female plasma was exposed to VAR2CSA expressing erythrocytes infected with the 3D7 strain. The plasma pool was sequentially exposed to four aliquots of MACS purified IE. In each step 2 × 10^−7^ μl of plasma per infected cell was used, corresponding to a total dilution of 5 × 10^−8^ μl plasma/IE. The histograms show IgG binding to infected erythrocytes stained with 5 μl plasma per 2 × 10^5^ cells. As secondary antibody 1.5 μg per 2 × 10^5^ IE of FITC conjugated goat-anti human IgG (Beckman Coulter IM1627) was used. Grey, blank control; red, female plasma pool (93% positive cells); blue, depleted female plasma pool (11% positive cells).
(1.7 MB TIF)

Table S1 Modeling Details of the VAR2CSA DBL Domains (32 KB DOC)

Table S2 Summary of Conserved Stabilizing Positions in Models of VAR2CSA DBL Domains (30 KB DOC)

## Abbreviations

aa: amino acid
CIDR: cysteine-rich inter-domain region
CSA: chondroitin sulphate A
DARC: Duffy antigen receptor for chemokines
DBL: Duffy-binding-like
DV: depletion value
EBA: erythrocyte binding antigen
IE: infected erythrocyte
ID2: inter-domain 2
PAM: pregnancy-associated malaria
PfEMP1: Plasmodium falciparum erythrocyte membrane protein 1
Pkα-DBL: Plasmodium knowlesi α-duffy-binding-like
S1–S3: sub-domains 1–3
