# Supplementary material for: Structural Insight into Epitopes in the Pregnancy-Associated Malaria Protein VAR2CSA
Source: PLoS Pathog. 2008 Feb 15;4(2):e42. doi: 10.1371/journal.ppat.0040042 (PMC2242842; doi:10.1371/journal.ppat.0040042)
Supplement: Supplementary file 1 [file ppat.0040042.st001.doc]

| Table S1. Modeling details of the VAR2CSA DBL domains | | | | | |
| --- | --- | --- | --- | --- | --- |
| VAR2CSA DBL domain | Modeling template | Template  sequence identity | Insertions relative to template | Missing info inserts* | Gaps relative to  templates |
| DBL1 | Pka-DBL | 16.4% | 10 | 4 | 2 |
| DBL2 | EBA-175 F1 | 17.2% | 10 | 1 | 0 |
| DBL3 | EBA-175 F1 | 20.7% | 6 | 1 | 1 |
| DBL4 | Pka-DBL | 17.8% | 9 | 4 | 1 |
| DBL5 | EBA-175 F1 | 18.8% | 8 | 1 | 2 |
| DBL6 | Pka-DBL | 18.7% | 8 | 4 | 1 |
| *Inserts in regions with missing structural information in the template structure. | | | | | |
