# Supplementary material for: Structural Insight into Epitopes in the Pregnancy-Associated Malaria Protein VAR2CSA
Source: PLoS Pathog. 2008 Feb 15;4(2):e42. doi: 10.1371/journal.ppat.0040042 (PMC2242842; doi:10.1371/journal.ppat.0040042)
Supplement: Supplementary file 2 [file ppat.0040042.st002.doc]

| Table S2. Summary of conserved stabilizing positions in models of VAR2CSA DBL domains | | | | |
| --- | --- | --- | --- | --- |
| VAR2CSA DBL domain | Buried hydrophobic | Buried polar/charged | Helix capping | Cysteines in positions of disulfide bridges* |
| Templates | 34 | 11 | 4 | 12(14) |
| DBL1 | 33 | 10 | 4 | 6 |
| DBL2 | 32 | 8 | 3 | 10 |
| DBL3 | 31 | 9 | 4 | 13 |
| DBL4 | 33 | 11 | 4 | 8 |
| DBL5 | 33 | 9 | 3 | 6 |
| DBL6 | 33 | 11 | 4 | 6 |
| *Counted in HHpred alignments used for modeling. The parenthesis denotes EBA175 F2. | | | | |
